# Anaesthesia-Related Pediatric Neurotoxicity: A Survey Study

**DOI:** 10.5152/TJAR.2022.21602

**Published:** 2022-04-01

**Authors:** Munise Yıldız, Betül Kozanhan, Eyüp Aydoğan, Yasin Tire, Tamer Sekmenli

**Affiliations:** 1 Department of Anaesthesiology and Reanimation, Health and Science University, Konya City Hospital, Konya, Turkey; 2 Department of Anaesthesiology and Reanimation, Bursa Gürsu State Hospital, Bursa, Turkey; 3 Department of Pediatric Surgery, Selçuk University Medical School, Konya, Turkey

**Keywords:** Induction of anaesthesia, intravenous agents, neurodevelopment, neurotoxicity, pediatric anaesthesia

## Abstract

**Objective:**

Millions of children are exposed to anaesthetic drugs every day; however, the possible adverse effects of these agents on the central nervous system remain controversial. This study evaluated anaesthesiologists’ and pediatric surgeons’ knowledge and daily practices regarding anaesthesia-induced neurotoxicity.

**Methods:**

A survey consisting of 12 questions was sent to members of the Turkish Anaesthesiology and Reanimation Association and the Turkish Pediatric Surgery Association via the Google forms program.

**Results:**

A total of 202 anaesthesiologists and 51 pediatric surgeons participated in this survey. The results demonstrate that anaesthesiologists and surgeons are aware of the risk of anaesthesia-related neurotoxicity and are willing to take action. Approximately, half of the anaesthesiologists and pediatric surgeons expected to postpone operations lasting at least 3 hours for patients <3 years of age. Also, one-third of the anaesthesiologists would seek feasible and more reliable alternative anaesthetic strategies.

**Conclusions:**

More than two-thirds of the participants knew about the US Food and Drug Administration neurotoxicity warning; however, uncertainty about anaesthesia-related neurotoxicity is ongoing. Many questions remain unanswered. The results of large-scale prospective randomized studies to evaluate the effect of anaesthetics and surgery on the cognitive development of pediatric patients are needed.

**Main Points**

- Anaesthesia-related neurotoxicity and how this issue impacts the clinicians’ perspectives and practical approaches is still a troubling unresolved dilemma.
- More than two-thirds of the participants knew about the US Food and Drug Administration neurotoxicity warning.

More than two-thirds of the pediatric surgeons stated that they did not operate on any infants in daily practice.

- Half of the participants preferred to delay operations expected to last for at least 3 hours beyond 3 years of age.

- The effects of anaesthesia and surgery on pediatric patients’ cognitive development are still an issue that needs to be evaluated.

## Introduction

Anaesthesia and sedation drugs are essential for infants and children undergoing surgery or other painful and stressful procedures. General anaesthesia and the use of anaesthetic agents were considered safe in pediatric patients until recently. However, many experimental studies and clinical data show that some anaesthetics that are used routinely in pediatric practice are not as harmless as they were once considered and may cause various adverse outcomes.^1-3^

Since 1999, preclinical studies have confirmed that exposing the developing brain to anaesthetic agents may cause neuronal apoptosis and neurodegenerative changes.^1-4^ Data from infant rodent and non-human primate studies show that exposure to general anaesthetic agents during the early postnatal period can produce cognitive, behavioral, and memory impairments later in life.^5^ Substantial evidence from preclinical studies has pushed researchers to investigate the toxic effects of anaesthesia, which may lead to long-term adverse neurodevelopmental outcomes in children. Mixed and conﬂicting results have been reported from human cohort studies.^6^ Several studies have demonstrated an association between an increased risk of adverse neurodevelopmental outcomes and exposure to anaesthesia in infants or toddlers,^7-10^ whereas other cohort studies have found no evidence for an association.^11-13^

The US Food and Drug Administration (FDA) issued a warning in 2016 stating the potential risks of repeated or prolonged anaesthesia exposure in infants, children, and pregnant women.^14^ However, the FDA warning was questioned by many editorials and medical societies due to insufficient and inconclusive clinical evidence.^15-17^ After strong responses, the FDA updated the advisory in 2017, stating that the label information described studies in young and pregnant animals and that a single, relatively short exposure was unlikely to have detrimental effects on behavior and learning.^18^ Therefore, surgeries or procedures in children <3 years and pregnant women should not be delayed or avoided when medically necessary, and practitioners should follow their usual practices.

Although the FDA safety warning is alarming, it is mainly based on preclinical study results. Recent well-controlled, prospective clinical studies indicate that a single exposure to general anaesthesia for <1 hour by infants does not alter long-term cognitive function.^19-21^ However, the effect of prolonged or repeated exposure to anaesthesia on the vulnerable brains of infants who require complex surgery, such as for coronary heart disease or a diaphragmatic hernia, is still unknown.

Every year, millions of children undergo various surgical and neuroimaging procedures worldwide. The increasing number of pediatric patients carries significant risks if delayed to an advanced age. However, concern about the risk of the development of neurotoxicity and the potential damage associated with canceling or delaying a required procedure should be weighed. Although concerns about the potential neurotoxic effects of the anaesthesia have increased, published clinical data contain crucial gaps regarding the decision for the optimal timing of elective procedures and whether specific anaesthetic agents and methods should be chosen to minimize risk. According to our knowledge and literature review, no similar study was performed in Turkey in this troubling unresolved dilemma. Therefore, we aimed to evaluate anaesthesiologists’ and pediatric surgeons’ knowledge and clinical practice, including obtaining informed consent from the parents, timing, and frequency of procedures regarding the possibility of anaesthesia-induced neurotoxicity in our country. 

## Methods

This survey study was carried out in accordance with the principles outlined in the Declaration of Helsinki between December 1, 2019, and December 30, 2019, after obtaining approval from the Non-Interventional Researches Ethics Committee of Selçuk University (decision number: 2018/353).

### Setting, Population, and Sample Size

This study was conducted by sending an online questionnaire to members of the Turkish Anaesthesiology and Reanimation Association and the Turkish Pediatric Surgery Association. The survey population consisted of 1202 anaesthesiology and reanimation specialists and 408 pediatric surgeons. With a sampling frame of 1610 subjects, 232 responses would be needed to obtain a representative sample with a 90% CI and a margin of error of ±5% (https://www.qualtrics.com/blog/calculating-sample-size/).

### Questionnaire Form

The survey consisted of 12 questions divided into 2 sections of knowledge and practice presented in Tables 1 and 2. The first 7 questions were to measure the knowledge level of the participants (Table 1). In the second section, questions 8 and 9 were directed to all participants, whereas questions 10-12 were prepared solely to assess the anaesthesiologists’ practical approaches regarding the possibility of neurotoxicity. We requested an agreement from participants at the beginning of the survey. The participants’ age, work experience, frequency of performing anaesthesia/surgical procedures in pediatric age groups, and opinions on anaesthesia exposure at an early age and informing parents about neurotoxicity were investigated. 

Ten anaesthesiology and reanimation specialists and 5 pediatric surgeons who routinely practice in the pediatric anaesthesia operating room completed a survey pretest regarding how long the survey should take to complete, comprehensiveness, and whether specific questions were ambiguous. 

### Data Collection Method

E-mail invitations and the online questionnaire forms were sent to members of their national associations with access to the survey link with an identification number and log-in code to complete the online survey. They were sent a second e-mail as a reminder after 2 weeks.

### Statistical Analysis

The statistical analysis was performed with IBM Statistical Package for the Social Sciences Statistics for Windows 10 version 22.0 software (IBM Corp., Armonk, NY, USA). Demographic data and the results of the multiple-choice questions are presented as frequencies and percentages. Responses for the Likert scale questions are given as frequencies and rates calculated as the number of positive or negative findings. 

## Results

Of the 1202 invited anaesthesiology and reanimation specialists, 240 responded to the survey (response rate = 19.9%); 38 indicated they were not involved in pediatric anaesthesia practice, resulting in 202 completed surveys for analysis. From 408 online survey requests of pediatric surgeons, 74 responses were received (response rate = 18%); 23 responses were excluded from the analysis (not working in a hospital), resulting in 51 completed surveys for analysis. 

The demographic data are shown in Table 3. The distribution of the age range of the participants was 35-55 years. Education and research hospitals as well as state hospitals constituted more than half of the institutions where participants worked. More than 80% of the responding pediatric surgeons had more than 10 years of work experience, while this rate was over 50% for the anaesthesiology and reanimation specialists.

The percentages of anaesthesiologists and pediatric surgeons who knew about the FDA neurotoxicity warning were 64.4% and 68.6%, respectively. 

**Figure 1.**
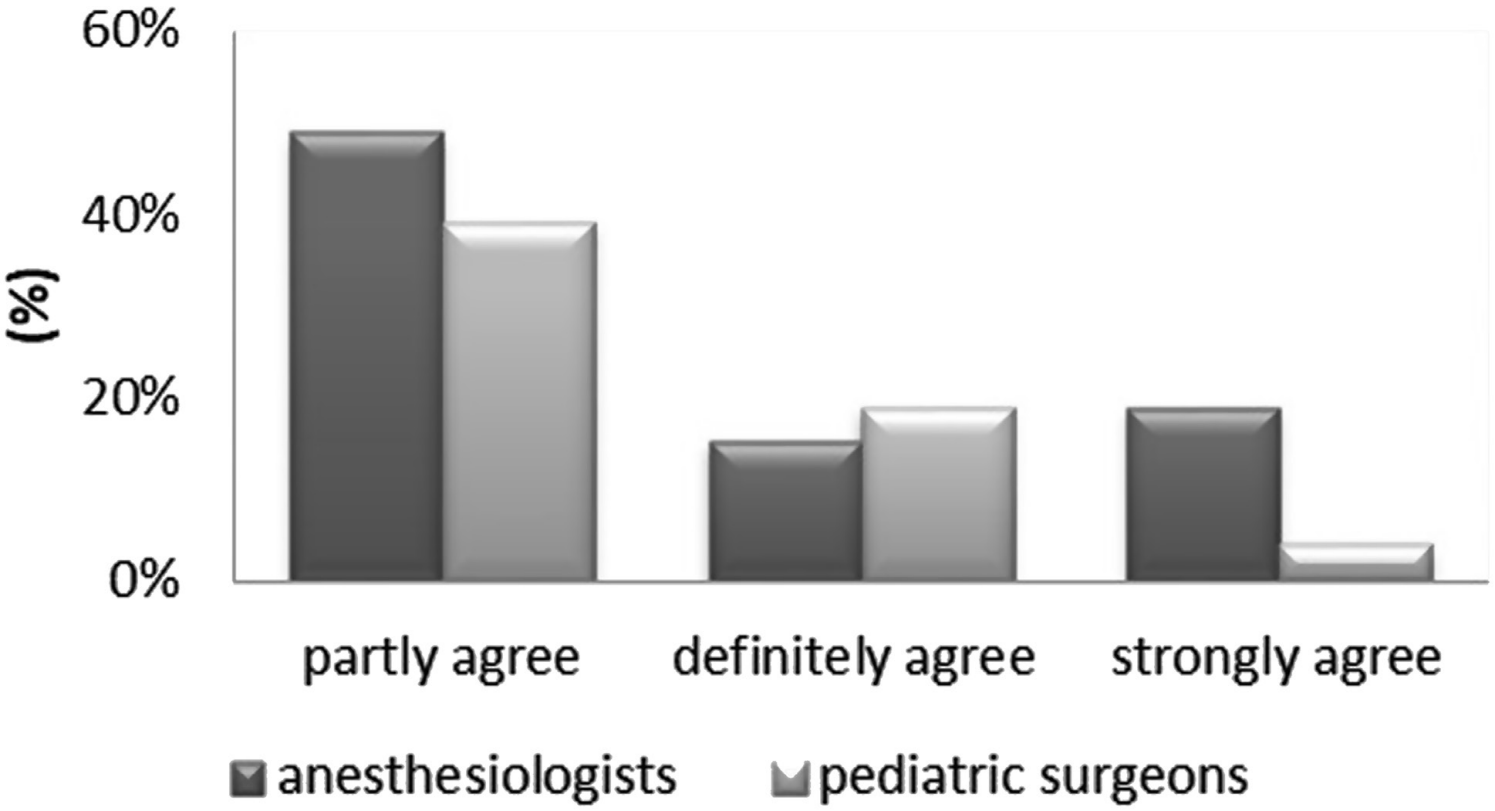
The relationship between early exposure to general anaesthetics and negative neurocognitive effects in children.

We investigated the participants’ opinion of an association between exposure to general anaesthetics at an early stage and adverse neurocognitive outcomes in question 2. In total, 19.3% of anaesthesiologists and 3.9% of pediatric surgeons disagreed strongly with this statement. About 49% of the anaesthesiologists and 39.2% of the pediatric surgeons partly agreed with this statement, and 15.3% of anaesthesiologists and 19.3% of the pediatric surgeons agreed with the statement (Figure 1).

**Figure 2.**
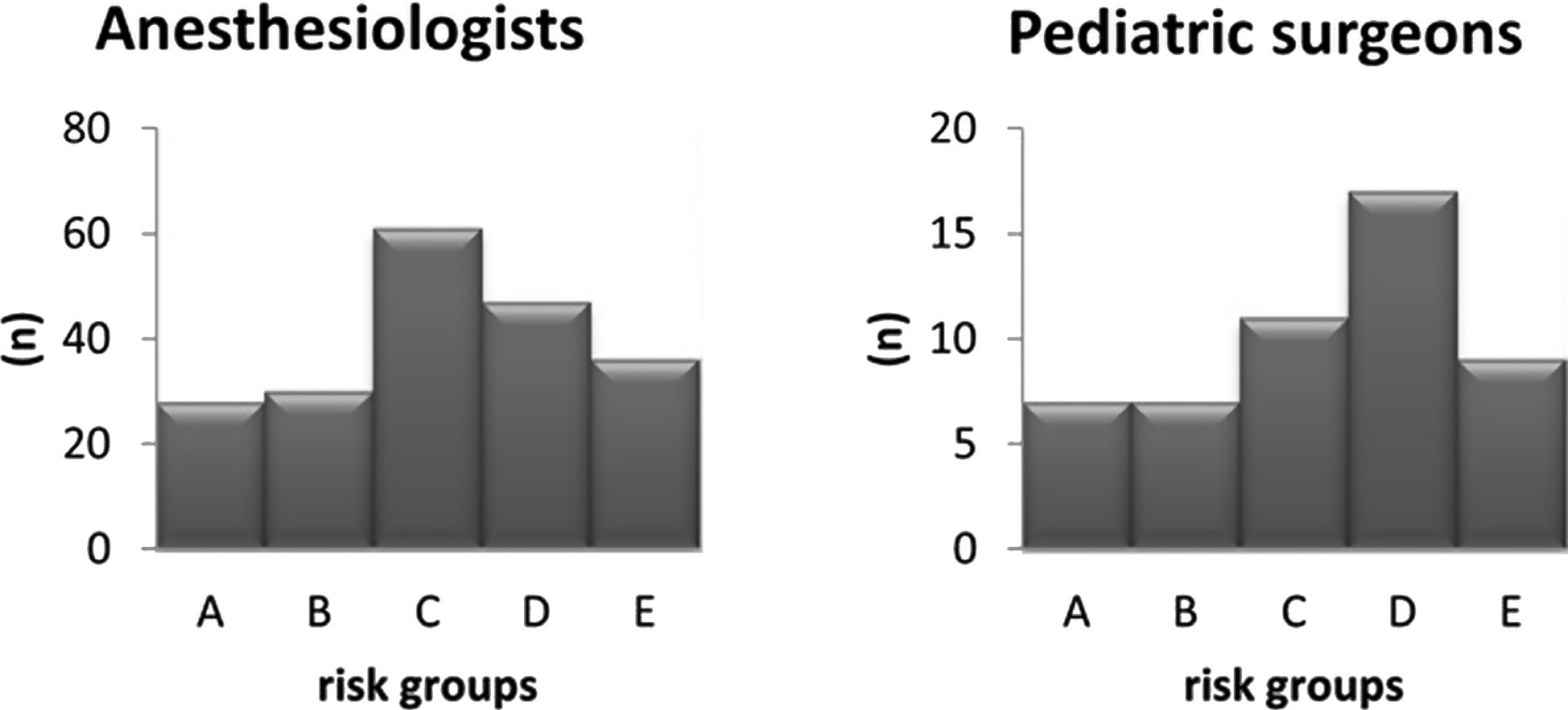
The knowledge of participants regarding groups of patients with increased risk of anaesthesia-related neurotoxicity. (A) Participants who chose only 1 of the high-risk patient groups. (B) Participants who chose 2 of the high-risk patient groups. (C) Participants who chose 3 of the high-risk patient groups. (D) Participants who chose 4 of the high-risk patient groups. (E) Participants who chose all of the high-risk patient groups.

In question 3, we aimed to investigate clinicians’ knowledge regarding children with an increased risk of anaesthesia-related neurotoxicity. We found that 4.5% of anaesthesiologists and 7.8% of pediatric surgeons chose the first option and stated that there was no increased risk for any age group exposed to anaesthesia. The number of the other options of the participants chose from option “b” to “f” was shown in Figure 2.

The percentages of anaesthesiologists and pediatric surgeons who suggested postponing operations after 3 years of age, in situations in which there were no expected additional complications in cases of delay, were 49% and 47.1%, respectively. 

The participants’ opinions about informing the parents of children <3 years of age undergoing surgery of the risk of anaesthesia-related neurotoxicity was investigated in question 5; 7.9% of pediatric surgeons and 6% of anaesthesiologists thought that there was no risk and no need to inform the parents; 9.8% of pediatric surgeons and 13.9% of anaesthesiologists preferred not to provide information to the parents due to unnecessary anxiety in the families; 5.8% of pediatric surgeons and 5.4% of anaesthesiologists thought that informing parents about the FDA warning should be done only at the parent’s request; 41.2% of the pediatric surgeons and 55.9% of the anaesthesiologist’s opinion was that clinicians should give information together. 

The opinions of the participants regarding the effect of additional factors, such as pain, stress related to surgery, and co-morbid diseases, on neurocognitive function in addition to anaesthesia exposure in children undergoing surgery, were analyzed in the 3 groups. The percentage of pediatric surgeons who thought that all of the additional factors had an impact on neurocognitive functions, other than exposure to anaesthesia, was 39.2%. In comparison, the rate among anaesthesiologists was 69.8%. About 28.7% of the anaesthesiologists and 53% of the pediatric surgeons stated that one or more factors could impact neurocognitive function. The ratios of pediatric surgeons and anaesthesiologists who did not choose any of these factors were 7.8% and 1.5%, respectively. 

About 91.6% of anaesthesiologists and 17.6% of pediatric surgeons stated that there is a consensus between anaesthetists and pediatric surgeons about safe age limits for the risk of anaesthesia-related neurotoxicity.

In the second section of the survey, we evaluated the frequency of applying anaesthesia/surgery of the participants for each sub-groups regarding patient age: preterm infants, newborns, patients aged 1-3, 3-6, and 6-18 years. The results of the analyses are presented in Tables 4 and 5. We also assessed the frequency of applying major and minor surgery rates in 1 month among participants. Two-thirds of the participants reported that they performed major surgery at least once a month (Tables 4 and 5). 

To reduce the risk of neurotoxicity associated with anaesthetics exposure, 91.1% of the participants considered changing their anaesthetic practice. These practical clinical changes are shown in detail in Table 6. Rates of preferring total intravenous anaesthesia or regional anaesthesia techniques instead of inhalation anaesthesia were 27.8% and 24.7%, respectively. We also investigated participants’ opinions on changing their practice from hypnotic-based anaesthesia to opioid-based anaesthesia techniques in premature babies; 75.2% of the anaesthesiologists stated that they did not prefer this in practice. 

## Discussion

In this survey study, we investigated anaesthesiologists’ and pediatric surgeons’ personal opinions about anaesthesia-related neurotoxicity and how this issue impacts their practice. The most significant result of the present study is that more than two-thirds of the participants knew about the FDA neurotoxicity warning. Also, approximately 80% of anaesthesiologists and 95% of pediatric surgeons had the opinion that exposure to general anaesthetics in children at an early age might have adverse neurocognitive effects in the future. Contrary to our results, Sedighinejad et al.^22^ evaluated physicians’ knowledge and practice in Iran regarding this issue and found that most participants did not believe in general anaesthesia toxicity. Similarly, Weber et al.^23^ assessed European pediatric surgeons’ thoughts and showed that only 25% of the respondents reported being concerned about neurotoxicity.

The FDA warning regarding anaesthesia-related neurotoxicity mainly covers children <3 years of age, due to rapid development of the fetal brain.^18^ Hence, most studies focused on infants and young children aged 1-3 months or 2-4 years, who are considered to be the most vulnerable to potential toxins during synaptogenesis of neurons during brain development.^5,7,10,24^ The results from epidemiological studies and prospective trials in human infants have failed to show adverse effects on cognitive development from a single anaesthetic episode of <1 hour.^19-21^ However, Weber et al.^23^ showed that about 40% of the European pediatric surgeons reported personal changes in their daily practice, including postponing truly elective procedures until age >2 years and discussing surgery indications with their colleagues. Similarly, in the present study, 66.8% of the pediatric surgeons stated that they did not operate any infants in daily practice. Although we cannot directly comment on whether this is related to the risk of anaesthesia-induced neurotoxicity, these preferences may cause severe clinical outcomes due to delayed surgery. 

Repeated and lengthy (>3 hours) exposure to anaesthetic agents may pose a risk for neurocognitive impairment in pediatric subjects as warned by the FDA.^18^ In the first large-scale retrospective study, Wilder et al.^25^ showed that the incidence of behavioral, learning, or developmental problems increases approximately 2-fold after prolonged or repeated exposure to anaesthesia when children have undergone anaesthesia before 4 years of age. About half of the anaesthesiologists and pediatric surgeons in Turkey preferred to delay operations expected to last for at least 3 hours beyond 3 years of age if no additional complications arose with the delay, similar to their counterparts in European countries.^23^

Collaboration between anaesthesiologists and surgeons may provide safe and effective care in the perioperative setting.^26^ Most of the anaesthesiologists (91.6%) thought that there was a consensus between anaesthesiologists and pediatric surgeons regarding safe age limitations for the risk of anaesthesia-related neurotoxicity. However, only 17.6% of pediatric surgeons thought similarly. 

Children who undergo surgery at an early age generally vary in significant ways from healthy children who do not. These confounding factors may play a role in the causal relationship between anaesthesia and developmental or behavioral consequences in children. Moreover, pain, stress, and poor cardiovascular, respiratory, and metabolic homeostasis are neurotoxic. Although retrospective studies have not investigated the effect of these confounding factors, most participants thought that additional factors might be an issue related to neurocognitive functions other than anaesthesia exposure in children undergoing surgery. Perioperative management should focus on mitigating confounding factors.

The FDA warning about informing parents of young children about the potential neurotoxic effects of anaesthetics may cause significant controversy among clinicians and the public since the literature is inconclusive.^27^ In the present study, 5.8% of pediatric surgeons and 5.4% of anaesthesiologists thought that informing parents about the FDA warning should be done only at the request of a parent, in line with Weber et al.^23^ findings. On the contrary, Ward et al.^28^ reported that 91% of the participants discussed the neurotoxicity only if parents asked. It remains controversial whether the preoperative information about anaesthesia increases parental anxiety.^29,30^ A small number of participants thought it was unnecessary to provide information regarding risk to prevent extreme family anxiety. 

When surgery is necessary, questions about how reliable anaesthesia can be applied and alternative neuroprotective strategies after prolonged exposure are still being investigated. Using a combination of dexmedetomidine, opioids, and a neuraxial block is advised as an alternative anaesthetic regimen to alleviate anaesthetic neurotoxicity in infants.^31^ Similarly, opioids are thought to be relatively benign in terms of apoptosis due to exposure to anaesthesia in the developing brain. Hence, Kuratani^32^ revealed that neonatal anaesthesia is changing from hypnotic-based anaesthesia to opioid-based anaesthesia to minimize the risk of neuroapoptosis. However, these alternatives are not feasible, particularly for short procedures and older children. In the present study, approximately one-third of anaesthesiologists were seeking more feasible and reliable alternative anaesthetic strategies. However, the majority of anaesthesiologists stated that they did not prefer high-dose opioid techniques. Similarly, European pediatric surgeons were reported to feel uneasy with this approach.^23^

The low response rate for this survey introduced a sampling bias, but, unfortunately, no consensus has been reached in many hospitals and associations on this subject. Therefore, the clinicians' perspectives and practical approaches discovered in our study may be valuable in terms of guidance for future studies. Our results require validation in a larger, more representative sample. Parents’ decisions can also be useful during the decision-making phase. Circumcision is usually performed at <1 year of age, and generally upon request of the parent. Parental choices were not questioned, which is another limitation of our study.

## Conclusions

The most important result of the present study is that more than two-thirds of the participants in both groups knew about the FDA neurotoxicity warning. However, there was no consensus between anaesthetists and pediatric surgeons for safe age limitations, delaying surgery, or informing parents about the risk of anaesthesia-related neurotoxicity. The uncertainty about anaesthesia-related neurotoxicity is ongoing, and there remain many questions to be answered in future studies, including: Do longer or repeat exposures have an effect on long-term cognitive performance? Which children are at the highest risk for anaesthesia-related neurotoxicity? At what age are children most vulnerable to anaesthetic agents? Should we inform the parents? Therefore, the results of large-scale prospective randomized studies to evaluate the effects of anaesthetics and surgery on the cognitive development of pediatric patients are needed.

## Figures and Tables

**Table 1.** The Knowledge of Participants

1-Have you ever heard of the FDA’s neurotoxicity warning? a. Yes b. No 2-Do you think that exposure to general anaesthetics at an early stage is associated with adverse neurocognitive effects in children? a. I strongly disagree b. Disagree c. No idea d. Partially Agree e. Absolutely agree 3-In which of the following patient groups do you think there is a risk of anaesthesia-related neurotoxicity? (Multiple options can be marked) a. I think there is no risk in any age group. b. I think there is a risk in premature babies. c. I think there is a risk in children 3 years and younger. d. I think there is a risk in children with additional comorbidity, such as congenital heart disease. e. I think there is a risk in children with long-term sedation in intensive care. f. I think there is a risk in children who have long-term, recurrent, and/or complicated surgical procedures. 4-Do you think that operations that are expected to last for at least 3 hours and which will not constitute an additional complication if postponed should be postponed after 3 years of age? a. No b. Yes 5-Should parents be informed about the risk of anaesthesia-related neurotoxicity to parents of children under 3 years of age? a. I don’t think there is such a risk and there is no need to inform. b. I think that this information will cause unnecessary anxiety in families and should not be informed. c. Information should be given only if the parent asks. d. Yes, information should be made by the anaesthesiologist for this risk in each interview. e. Yes, the information should be made by the anaesthesiologist and the surgeon at each interview. f. Yes, information should be made by the surgeon for each risk. 6-What other factors do you think are effective on neurocognitive functions other than anaesthesia exposure in children undergoing surgery? a. Pain b. Stress related to surgery c. Comorbid diseases such as cardiovascular, respiratory, and/or metabolic diseases d. All e. None 7-Do you think there is consensus among anaesthesiologists and pediatric surgeons about safe age limitation for the risk of anaesthesia-related neurotoxicity? a. Yes b. No

Questions from 1-7 were directed to both anaesthesiologists and pediatric surgeons.

**Table 2.** The Practice of Participants

8-What is your frequency for applying pediatric anaesthesia/surgery for each sub-groups regarding patient age? (Please select pediatric anaesthesia/surgery frequency for each age category below from “none/less than 5 per month/5-10 per month/more than 10 per month”; preterm infant; newborn; 1-36 months; 3-6 ages; 6-18 age) 9-The frequency of anaesthesia/surgery for the following types of pediatric surgeries? a. Major surgery (defined as cardiac, orthopedic, neurosurgical, oncologic surgeries, etc.); none/less than 5 per month/5-10 per month/more than 10 per month b. Minor surgery (defined as circumcision, hernia repair, adenoidectomy-tonsillectomy, etc.); none/less than 5 per month/5-10 per month/more than 10 per month 10-Do you make any changes to your practical applications to reduce the risk of neurotoxicity associated with anaesthetics? a. No b. Yes 11-In order to reduce the risk of neurotoxicity associated with anaesthetics, how did you change your practice and/or consider doing it? a. I prefer regional anaesthesia techniques instead of general anaesthesia. b. I prefer TIVA instead of inhalation anaesthesia. c. I talk to the surgeon and the family to postpone the operation until a certain age. d. I prefer agents such as Remifentanil and dexmedetomidine. e. I do not think of any changes. 12-Would you prefer the method of anaesthesia “using only opioids and muscle relaxants, without hypnotics” a technique that has recently become increasingly popular, in premature babies? a. I never used this technique. b. No, I do not prefer. c. Yes, I often use this technique.

Questions 8 and 9 were directed to both anaesthesiologists and pediatric surgeons; however, questions from 10 to 12 were only directed to anaesthesiologists.

TIVA, total intravenous anaesthesia.

**Table 3.** Physicians’ Demographic Characteristics

	Pediatric Surgeon, n (%)	Anaesthesiologist, n (%)
**Age (years)**		
25-34 35-44 45-54 More than 55	3 (5.8) 22 (43.2) 17 (33.4) 9 (17.6)	36 (17.9) 112 (55.4) 46 (22.8) 8 (3.9)
**Years of experience**		
Less than 5 5-10 10-20 More than 20	1 (2) 8 (15.6) 23 (45.1) 19 (37.3)	15 (7.5) 71 (35.1) 96 (47.5) 20 (9.9)
**Institution**		
State hospital Training and research hospital University hospital Private hospital Gynecology and children’s hospital	25 (49) 11 (21.6) 6 (11.8) 2 (3.9) 7 (13.7)	57 (28.2) 83 (41.1) 34 (16.8) 1 (0.5) 27 (13.4)

**Table 4.** Major and Minor Surgery Rates and Frequency for Applying Pediatric Anaesthesia/Surgery per Month for Each Group Regarding Patient Age Among Anaesthesiologists

Case number per month, n (%)	0	1-4	5-10	≥10
**Classification of age**				
Preterm infant Newborn 1-36 months 3-6 years 6-18 years	69 (34.1) 84 (41.6) 50 (24.8) 28 (13.9) 27 (13.4)	25 (12.4) 47 (23.3) 79 (39.1) 75 (37.2) 68 (33.6)	99 (49) 33 (16.3) 9 (4.5) 6 (2.9) 5 (2.5)	9 (4.5) 38 (18.8) 64 (31.6) 93 (46.0) 102 (50.5)
**Type of surgery**				
Major surgery Minor surgery	67 (33.2) 16 (7.9)	32 (15.8) 67 (33.2)	84 (41.6) 8 (4.0)	19 (9.4) 111 (54.9)

**Table 5.** Major and Minor Surgery Rates and Frequency for Applying Pediatric Surgery per Month for Each Group Regarding Patient Age Among Pediatric Surgeons

Case number per month, n (%)	0	1-4	5-10	≥10
**Classification of age**				
Preterm infant Newborn 1-36 months 3-6 years 6-18 years	34 (66.8) 12 (23.6) 3 (5.9) 5 (9.8) 3 (5.9)	6 (11.7) 18 (35.3) 18 (35.3) 16 (31.4) 18 (35.3)	9 (17.6) 1 (1.9) 1 (1.9) 0 (0.0) 0 (0.0)	2 (3.9) 20 (39.2) 29 (56.9) 30 (58.8) 30 (58.8)
**Type of surgery**				
Major surgery Minor surgery	17 (33.3) 1 (1.9)	17 (33.3) 7 (13.7)	2 (3.9) 0 (0.0)	15 (29.5) 43 (84.4)

**Table 6.** Anaesthesiologist Preference for Making Changes in Practical Applications to Decrease the Risk of Neurotoxicity

	Anaesthesiologist, n (%)
**Changes in practical applications**	
Preferring regional anaesthesia Preferring TIVA Preferring to postpone the operation until a certain age Preferring remifentanil and dexmedetomidine Preferring not to change the protocol	50 (24.7) 56 (27.8) 39 (19.3) 39 (19.3) 18 (8.9)

TIVA, total intravenous anaesthesia.
